# Epidemiology of Femur Fractures in Children: A Descriptive Cross Sectional Study Based on a Rural Population of Nepal

**DOI:** 10.31729/jnma.5091

**Published:** 2020-08-31

**Authors:** Poojan Kumar Rokaya, Dhan Bahadur Karki, Mangal Rawai, Deoman Limbu, Bishnu Dutta Acharya, Pratap Babu Bhandari

**Affiliations:** 1 Department of Orthopedics & Trauma Surgery, Karnali Academy of Health Sciences, Jumla, Karnali, Nepal; 2 Department of Orthopedics, Chitwan Medical College, Chitwan, Nepal

**Keywords:** *children*, *epidemiology*, *femur*, *fracture*, *rural*

## Abstract

**Introduction:**

Femur fracture in children is one of the most common lower limb fractures which require inpatient care. The aim of this study is to determine the epidemiology of femoral shaft fractures in children from a rural population of Karnali Nepal.

**Methods:**

Hospital records were retrospectively reviewed from May 2017 to April 2020 to identify all the children with femur fracture. Sociodemographic profile, mode of injury, fracture pattern and location, time of presentation, initial treatment by traditional bone setters, treatment method and duration of hospital stay were noted. Data analysis was done using Statistical Package for Social Sciences version 20.

**Results:**

Altogether 104 children were identified. The mean age was 5.55 years and boys predominated 65 (62.5%). Falls were the major mode of injury in 65 (62.4%) patients. Fractures were frequently noted between April 15 to August 15. There were four (3.8%) open fractures and concurrent fracture observed in eight (7.6%) patients. Ten (9.6%) children received prior treatment from traditional bonesetters. Treatment methods included hip spica 62 (59.6%), elastic intramedullary nailing 30 (28.8%) and plate fixation 12 (11.5%). The duration of hospital stay in the nailing and plate fixation group was 11.43 days and 18 days respectively

**Conclusions:**

Fracture was common in 2-6 years of age group in boys during summer. Fall from cliff, rooftop and ladder were the major preventable cause of fracture. Delayed presentation and prior treatment with traditional bone setters add special challenges to orthopedic surgeons working in rural teaching hospital.

## INTRODUCTION

Femoral shaft fracture in children is one of the most common lower limb fractures requiring inpatient admission. They represent 1.6 % of all fractures in children.^1^ At present, we treat children below six years with one and a half spica with or without prior traction. For children above six years treatment depends upon child's age, body habitus, fracture personality and location, concurrent injuries, skin condition and socioeconomic factors. Available options include elastic intramedullary nailing (EIN), external fixation and osteosynthesis with dynamic compression plate and screws.

Various studies on cause, pattern and treatment methods of childhood femur fractures have been conducted in urban settings of developed countries.^2–5^ To our understanding, epidemiological study on childhood femur fracture has not been conducted in rural population of Nepal till date. Our teaching hospital is situated in himalayan mountainous district Jumla of Karnali province. It is the health referral center of adjoining four districts Kalikot, Mugu, Dolpa and Humla which extends from 2500 to 4000 meters in elevation and inhabits 426,026 people.^6^ Karnali province is one of the remotest and most deceived regions of Nepal. Karnali has literacy rate of 62.7% and the lowest human development index 0.445 as compared to other parts of Nepal.^7^

The main objective of this study is to determine the clinical sociodemographic parameters of femoral shaft fractures and to review the treatment methods in children from rural population of Karnali Nepal.

## METHODS

A descriptive cross sectional single centre study was carried out at Karnali Academy of Health sciences Jumla, Nepal after ethical approval from Institutional Review Committee (IRC KAHS Reference; 35/076/077). Hospital inpatient records, operation notes and radiological charts were retrospectively reviewed from May 2017 to April 2020 to identify all children with femoral shaft fractures. Patient sociodemographic profile, mode of injury, fracture pattern and location, concurrent injuries, nerve and vascular injury, time of presentation, initial treatment by traditional bone setters, treatment method and length of hospital stay (LOHS) were recorded in patient proforma. Children from birth to 14 years of age admitted for femoral shaft fracture with complete medical records were included in the study. Exclusion criteria included: children above 14 years, inter trochanter or neck of femur fracture, femur growth plate injury, intra-articular fracture and patient with incomplete clinical details.

Initially children with femoral shaft fracture were kept in Buck's traction with < 2.5 Kg weight. Some children were referred in from others centers with immobilization in above knee posterior plaster slab. Duration of traction was determined by patients age, fracture pattern, initial shortening of more than two cm and skin condition. Hip spica was applied under appropriate analgesia after fracture reduction by orthopedic surgeons. Spica incorporated the lower abdomen, pelvis, thigh and leg of injured side and thigh of contralateral side with cross bar in between. The optimal position of hip spica includ ed bilateral hip abd uction of 30°, hip and knee flexed 30-45° and leg in 15° of external rotation. X-Ray of the affected thigh was obtained to assess reduction. Children with spica were discharged the following day. Danger signs of plaster and spica care were advised to the parents. Open fractures were managed with wound debridement, intravenous antibiotics and skeletal traction.

Use of EIN or plate fixation was dependent on patient's age, fracture pattern and location. EIN was performed using a standard technique. Patient was placed in a fracture table and retrograde nailing was done protecting the distal femoral physes. Nail size was calculated from 40 % of narrowest femoral medullary canal. Closed reduction was attempted under fluoroscopic guidance. Mini open reduction with minimal periosteal stripping was done when closed reduction failed upon three to four attempts. Affected extremity was immobilized in above knee posterior plaster slab. Check X-Ray was ordered on the first postoperative day.

Unstable femoral shaft fracture involving the distal or proximal third in patient older than six years were fixated with plate and screw. In supine position through direct lateral approach, fascia lata and vastus lateralis were incised to reach the fracture site. Fracture was reduced using bone clamps and fixated with narrow compression plate and screws. Three to four screws were placed on either side of fracture. Hemostasis achieved, wound closed in layers after irrigation with normal saline. Plaster slab immobilization was not used in this cohort. Patients were advised for gentle range of motion of ankle and knee as pain tolerated. Operative procedure was performed by council registered orthopedic surgeons. All the relevant data were collected and entered in patient proforma. Descriptive analysis of the data was done using Statistical Package for Social Sciences (SPSS Inc. version 20, Chicago, Illinois) to find out the number, percentage, mean, standard deviation, average and range.

## RESULTS

**Figure 1.**
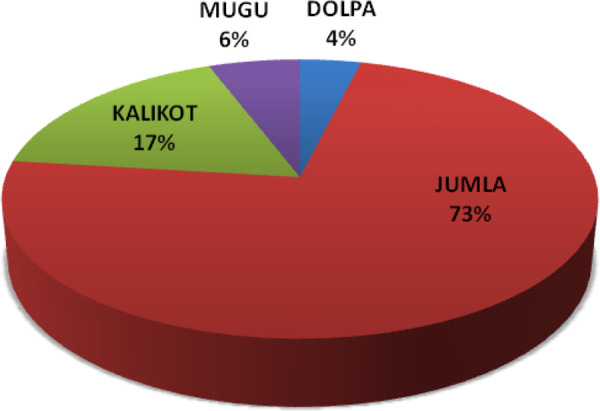
Geographic distribution of patients.

Altogether 104 children were admitted for femoral shaft fractures over a period of 3 years. The geographical distribution of patients is shown (Figure 1). A total of 28 (27%) children were primarily managed in other districts and were referred in cases.

**Figure 2.**
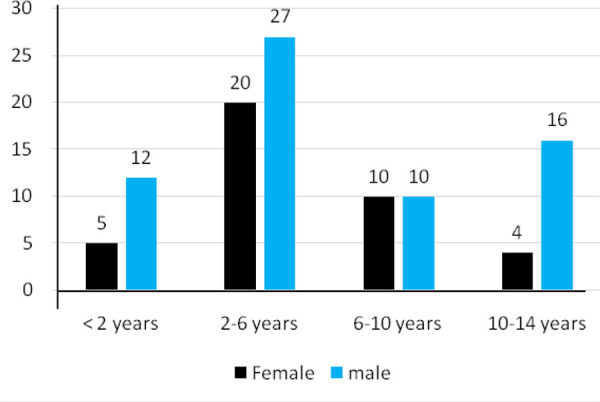
Number of fracture by age group.

There were 65 (62.5%) boys and 39 (37.5%) girls. The mean age was 5.55 years (8 months -14 years). Fracture was prevalent in 2-6 years of age group as mentioned (Figure 2).

Patient clinical demographic profile is presented (Table 1).

**Table 1:** Clinical demographic profile of patient.

	Variables	n (%)
Gender	Boys	65 (62.5)
	Girls	39 (37.5)
Side	Right	46 (44.2)
	Left	58 (55.8)
	Fall from ladder	26 (25)
	Fall from rooftop	23 (22.1)
	Fall from cliff	16 (15.3)
Mode of injury	Sports	13 (12.5)
	Road traffic accidents	10 (9.6)
	Fall from bicycle	9 (8.6)
	Physical assault	4 (3.8)
	Fall from horse	3 (2.8)
Fracture type	Open	4 (3.8)
	Close	100 (96.1)
Concurrent fractures	Mild head injury Ipsilateral distal radius	
	physeal injury	3 (2.8)
	Pubic Rami fracture	1 (0.9)
	Ipsilateral Ring and little	1 (0.9)
	finger phalanx fracture	1 (0.9)
	Contralateral Iliac Blade	1 (0.9)
	fracture	1 (0.9)
	Contralateral hemithorax	
	6-8 rib fracture	

**Figure 3.**
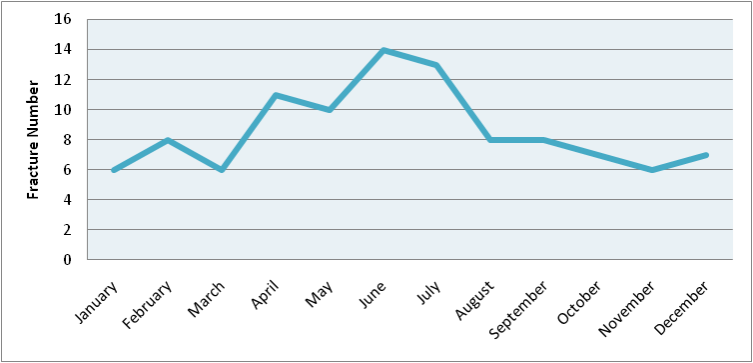
Seasonal distribution of femur fracture.

The seasonal pattern of fracture is shown (Figure 3). Right extremity was involved in 46 (44.2%) and left extremity in 58 (55.8%). Fall from cliff, ladders and rooftops were the major mode of injury accounting for 65 (62.4%) falls. Fracture pattern and location are summarized (Table 2).

**Table 2:** Fracture pattern and location.

		n (%)
Fracture Pattern	Transverse	37 (35.5)
	Short oblique	16 (15.3
	Long oblique	13 (12.5
	Spiral	22 (21.1)
	Comminuted	10 (9.6)
	Undisplaced	6 (5.7)
Fracture Location	Proximal Third	19 (18.2)
	Middle Third	68 (65.3)
	Distal Third	17 (16.3)

There were 100 (96.1%) closed fractures and four (3.8%) open fractures. Concurrent fracture was noted in eight (7.6%) patients. Ten out of 104 (9.6%) children were initially managed by traditional bonesetters and faith healers.

Average duration between day of injury and day of presentation to hospital was 3.11 days (Range 1-8 days). Treatment method with average LOHS is tabulated (Table 3). We did not notice bilateral fracture, neurovascular injury and mortality related to femur fracture.

**Table 3:** Treatment method with LOHS.

	n (%)	LOHS days Mean ± S.D.(Range)
Hip spica	62 (59.6)	7.52 ± 4.37 (2-16)
EIN	30 (28.8)	11.43 ± 4.74 (5-19)
Plate fixation	12 (11.5)	18 ± 3.33 (11-22)

## DISCUSSION

This study reflects the epidemiological pattern of childhood femoral shaft fracture treated in a rural teaching hospital of Karnali Nepal. The mean age of patient was 5.55 years with common occurrence in 2-6 years of age group. Our finding with respect to age distribution is in contrast to the study conducted by Hinton et al. where they observed bimodal age distribution.^8^ Male outnumbered female with 1.6:1 male to female ratio which is comparable with study conducted by Bridgman et al.^9^ Boys are more commonly involved in risk talking outdoor activities and sports as compared to girls which could relate this gender discrepancy. Fracture was common in summer and spring from April 15 to August 15. Outdoor activities and sports events are more likely in the warm months. Some studies have cited seasonal variation in fracture pattern as a result of increased activities in summer due to school vacation.^4–5^ Fall from ladder, rooftop and cliff were the frequent cause of injury observed in this study accounting for 65 (62.4%) of total falls. This finding differs with epidemiological studies from developed countries with larger series where fall from less than two meters was the predominant cause of injury.^3,10^ Children occasionally climb cliff to cut grass for household purpose in highland of Jumla, Kalikot, Mugu and Humla. People have compulsion to walk in the uneven trails of hills and mountain for their day to day agricultural livelihood. In addition, rooftops are usually open and lack protective bars or railings in most of the rural community of Karnali Nepal. Eight children (7.6%) with femoral shaft fracture had multiple injuries. Associated injuries in pediatric femoral shaft fracture have been reported up to 8.1% in the literature.^10^ Multiple injuries in a child with femoral shaft fracture tend to prolong the hospital stay and influence the existing treatment methods.^11^ We found 100 (96.1%) closed and 4 (3.8%) open femoral shaft fracture. A similar rate of open femur fracture is mentioned in other epidemiological studies.^10,11^ Majority of the fractures 68 (65.3%) were located in the middle third whereas 36 (34.5%) of the fractures were located in the proximal and distal third of femoral shaft. This is in accordance with other studies which revealed similar location of fracture.^12^’^13^ Average duration between time of injury and time of presentation to hospital was 3.11 days. Delay in presentation is a common scenario in remote health facilities of Karnali because of lack of road access, uneven geographical topography and initial management by traditional bonesetter and faith healers. Absence of road connectivity to all the remote village and shortage of ambulance compels patients to be carried in bamboo basket or stretcher for several days to reach the hospital. In this study, 10 (9.6%) out of 104 patient received prior treatment with traditional bonesetters and faith healers. Lack of awareness, poverty, negligence by the parents to seek medical advice and unavailability of nearby health facility could have promoted faith healers and traditional bone setters in rural areas of lower middle income country like Nepal.^14^

Our hospital treatment protocol for childhood femoral shaft fracture is individualized on age, body habitus, fracture stability and location, skin condition and associated injures. Among 104 children, 62 (59.6%) were treated with one and a half hip spica. Hip spica is an accepted treatment method for closed isolated femur fracture in children less than six years. Our treatment method of hip spica with respect to age less than six years is supported by various studies which have advocated immediate to early hip spica for the treatment femoral shaft fractures in children.^15–17^ Till date we are applying one and a half hip spica and have no experience with single leg walking hip spica. Few authors have found single leg walking hip spica to be superior and effective compared to traditional spica casting.^18,19^

Thirty children were managed with EIN. We use EIN for central 2/3 diaphyseal fracture, length stable fracture (transverse, short oblique) in children older than six years for internal fixation of femur fracture. Our current practice is compatible with systemic review and meta-analysis of 1012 patients conducted by Imam et al. who preferred EIN for the treatment of femur fracture in children aged less than 16 years.^20^ Although EIN is ideal for length stable fracture they have been equally effective in length unstable femoral shaft fracture in children less than 11 years.^21,22^

Osteosynthesis with dynamic compression plate and screws was used to treat 12 (11.5%) femoral shaft fractures. Plating is usually preferred in older children with unstable femoral fracture at the proximal and distal third where nailing can be technically difficult.^23^ Plating can achieve excellent stability but at the cost of bleeding, infection and scarring. Plating was done in the conventional manner as per the operating surgeons experience and preference. Minimal invasive submuscular plating has become a viable option for the treatment of length unstable femoral fracture in older and obese children.^24^

There has been a decreasing trend in duration of hospital stay in pediatric femur fracture managed operatively in the developed countries where they have standard and accessible health facility. The duration of hospital stay in the EIN and plate fixation group was 11.43 days and 18 days respectively which is contradictory to the western literature.^25,26^ Patient reside in the remote parts where they may not have health facility to remove the sutures during postoperative period. Parent's willingness to get their child discharged after suture removal usually on 12 to 14 postoperative days lengthens the hospital stay in our setup.

Retrospective nature of the study with small sample size is one of the limitations of this study. We could not comment on fracture union, complications and outcome as this study was intended to focus on epidemiological parameters of fracture. Study was conducted on patients presenting to one of the rural health referral center of Nepal which does not represent all children of Nepal. Multi centric study should be conducted in other rural health care centers to consolidate our results.

Based on our findings various activities to consider with priorities are 1) improve structural design of rooftops and ladders in rural areas 2) promote research on preventable injuries 3) campaign for awareness in the rural community regarding preventable injuries 4) counsel faith healers and traditional bone setters on their perception towards injured patients 5) strengthen the existing health referral system 6) access to specialized health service in rural parts of country.

## CONCLUSIONS

Femur fracture was common in 2-6 years of age in boys during summer. Fall from cliff, rooftop and ladder were the major preventable cause of fracture. Delay in presentation and initial treatment by traditional bone setters add special challenges to orthopedic surgeon working in rural teaching hospital.
